# Elucidation of the thermo-kinetics of the thermal decomposition of cameroonian kaolin: mechanism, thermodynamic study and identification of its by-products

**DOI:** 10.1039/d5ra05149e

**Published:** 2025-09-08

**Authors:** Cyrille Ghislain Fotsop, Alexandra Lieb, Franziska Scheffler

**Affiliations:** a Otto-von-Guericke-University Magdeburg, Chemical Institute, Chair for Industrial Chemistry Universitätsplatz 2 39106 Magdeburg Germany franziska.scheffler@ovgu.de

## Abstract

This work elucidates the thermo-kinetics of the thermal conversion of cameroonian kaolin to metakaolin as the main product. The thermokinetical parameters (activation energy *E*_a_ and pre-exponential factor *A*) for the kaolin conversion were calculated using model-free methods, *i.e.* the Kissinger–Akahira–Sunrose (KAS) and the Flynn–Wall–Ozawa (FWO) method, and differential methods (Kissinger and Ozawa) additionally including iterative procedures for KAS and FWO methods (KAS-Ir; FWO-Ir). The cameroonian kaolin was heat-treated using three different heating rates, *i.e.* 5, 20 and 40 K min^−1^, leading to metakaolin samples named MK-(5), MK-(20) and MK-(40). The TGA analysis showed a total mass loss of ∼12.5% in two steps related to the dehydration (step 1) and dehydroxylation (step 2). The *E*_a_ of the two steps were most accurately determined using the iterative procedures KAS-Ir and FWO-Ir. The average *E*_a_ values were 88.44/88.58 kJ mol^−1^ for step 1 and 261.85/261.91 kJ mol^−1^ for step 2, for the KAS-Ir and FWO-Ir models, respectively. The most probable mechanism function was determined by the multiple heating rate method (MHR) and the Coats-Redfern method. The kinetic analyses showed that the dehydroxylation of kaolin is controlled by a random nucleation and subsequent growth mechanism (G_4_) and a second order chemical reaction (F_2_). Thermodynamic parameters, namely the entropy Δ*S*^≠^, the enthalpy Δ*H*^≠^ and Gibbs free energy Δ*G*^≠^, were evaluated. The average values of Δ*S*^≠^, Δ*H*^≠^ and Δ*G*^≠^ using both the KAS-Ir and FWO-Ir models exhibited less than 5% deviation. The obtained metakaolin samples were characterized using X-ray diffraction (XRD), field emission scanning electron microscopy (FE-SEM) and Fourier-transform infrared spectroscopy (FT-IR).

## Abstract 2

This work elucidates the thermo-kinetics of the thermal conversion of cameroonian kaolin to metakaolin as the main product.

## Graphical abstract

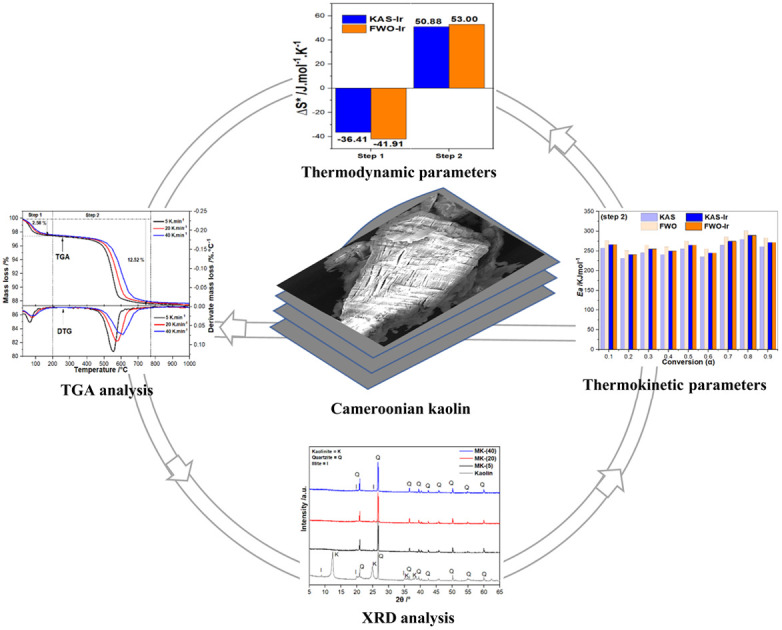

## 1 Introduction

Most of the natural or synthetic clay minerals are used as additives or raw materials for the manufacturing processes of essential products.^1^ Smectites, vermiculites, kaolin and sepiolites are among the most abundant and widely used clay minerals in pure or crude forms.^2,3^ Due to their various physicochemical properties, mineralogical compositions and colors^4,5^ they have various applications in chemical, petrochemical, paper, pharmaceutical, plastic and cosmetic industries.^6^ Kaolin, mainly containing kaolinite Al_2_Si_2_O_5_(OH)_4_, is a phyllosilicate consisting of layers of corner sharing silicate tetrahedra [SiO_4_] and sheets of edge sharing aluminum octahedra [AlO_3_(OH)_3_].^2^ Kaolinite is one of the most important raw materials used in the porcelain industry.^7^ The mineral composition (46.54% SiO_2_, 39.50% Al_2_O_3_ and 13.96% H_2_O) of kaolinite and other phases such as quartz, illite, feldspars or muscovite^8^ allows kaolin to have applications in the cement industry and in the preparation of geopolymers, paints and dyes.^9,10^ Additionally, the thermal activation of kaolin between 450 and 850 °C, ultrasonic treatment^11,12^ and mechanical treatment^13,14^ facilitate the delamination of the structure by breaking down the ordered crystalline structure of kaolinite and promoting the formation of a highly reactive amorphous material, namely metakaolin.^15^ Metakaolin is widely used for the production of zeolites and geopolymers.^16,17^ Knowledge of the mechanisms and processes that occur during the thermal activation of kaolin to metakaolin is crucial to understand the transformation of metakaolin to zeolites.^18^ The thermal treatment of kaolin is a complex process, because the chemical stoichiometry of the initial phases and their exact hydration state is often unknown.^19–21^ The kinetics and the mechanism of kaolin conversion is mostly affected by the heating rate^22^ and the partial pressure of water vapor.^23,24^ Chen *et al.*, 2004, reported a sequence of processes during the thermal conversion of kaolin, such as an endothermic dehydroxylation step and the conversion of kaolin to metakaolin in the temperature range from 450 to 700 °C, the formation of amorphous silica and cubic spinel phases at around 950 °C, the formation of stable mullite phases by an exothermic reaction at around 1100 °C and the crystallization of amorphous silica to cristobalite at temperatures above 1100 °C.^23,24^ However, this sequence does not give deeper insights in the decomposition kinetics, the thermodynamic properties and the controlling mechanism of the dehydroxylation of kaolin. The thermokinetics are still poorly understood, due to the complex diffusion phenomena of the water molecules in kaolin.^25^ Thermal analysis techniques, such as differential scanning calorimetry (DSC),^26^ thermogravimetric analysis (TGA)^13^ and differential thermal analysis (DTG and DTA)^27^ are the most commonly used methods for the assessment of dehydroxylation processes of kaolin.^19^ These techniques coupled with IR and Raman spectroscopy and X-ray diffraction allow confirmation of the formation of metakaolin.^28^

Several related studies on kaolinite dehydroxylation and kinetics have been reported in the literature.^29,30^ However, the determination of accurate values for *E*_a_ and *A* remains a challenge. The reported work^30–34^ highlights the determination of *E*_a_ and *A* of the dehydroxylation processes of kaolin based on TGA analyses and derived DTG and DTA curves, using Ozawa, Kissinger, Starink, Flynn–Wall–Ozawa (FWO), Kissinger–Akahira–Sunrose (KAS) equations and the Coats-Redfern model. Recently, Irfan Khan *et al.*, 2017 ^29^ reported on the pyrolytical conversion of kaolin to metakaolin based on TGA, DTG and DTA analyses. The authors showed that the step controlling the mechanism of conversion and kinetics was a third-order chemical reaction (F_3_). Furthermore, higher values of *E*_a_ were found using integral models of KAS, Starink and FWO compared to the values from differential methods. Ptáček *et al.*, 2014 also reported on the thermokinetics of the kaolin to metakaolin conversion based on TGA data using various heating rates, concluding that the process of conversion is controlled by delamination, dehydroxylation and subsequent recrystallization of alumina and silica tetrahedra.^35^ To the best of our knowledge, the thermodynamic parameters, such as the entropy Δ*S*^≠^, the enthalpy Δ*H*^≠^ and Gibbs free energy Δ*G*^≠^ and the determination of accurate values for *E*_a_ using an iterative procedure by KAS-Ir and FWO-Ir methods, were not yet investigated.^36^

Our work investigates the major thermokinetical parameters *E*_a_ and *A* of the conversion of kaolin based on TGA data and DTG curves, using an iterative procedure by applying the FWO-Ir and KAS-Ir models. Subsequently, the thermodynamic parameters Δ*S*^≠^, Δ*H*^≠^ and Δ*G*^≠^ and the most probable mechanism function controlling the thermal conversion of kaolin, using the Coats-Redfern model and MHR method, were determined. Scheme S1 gives an overview of the sequence of the kinetics investigation. Finally, the derived product were subsequent characterized using X-ray diffraction (XRD), field emission scanning electron microscopy (FE-SEM), Fourier-transform infrared (FT-IR) and Raman spectroscopy analysis.

## 2 Materials and experimental procedure

### 2.1 Materials

The raw kaolin material was collected in the West Region of Cameroon (Fig. S1). Its chemical composition was determined by X-ray fluorescence analysis (XRF) showing a molar Si/Al ratio of 2.6 (Table 1).

**Table 1:** Chemical composition of cameroonian kaolin determined by XRF analysis

Oxide	SiO_2_	Al_2_O_3_	CuO	K_2_O	H_2_O	P_2_O_5_	CaO	TiO_2_	MnO	ZrO_2_	Fe_2_O_3_
Weight%	52.02	34.01	0.006	0.441	12.90	0.160	0.011	1.201	0.002	0.012	0.545

### 2.2 Experimental procedure: thermal treatment of cameroonian kaolin

Prior to all of the TGA measurements the raw kaolin was dried in an oven at 100 °C overnight and then stored in a desiccator over silica gel. Thermogravimetric analyses, each with ∼1.5 g of kaolin, were performed at heating rates of 5, 20 and 40 K min^−1^ in a temperature range from 25–1100 °C. The achieved metakaolin samples were labeled MK-(*x*), where *x* is the heating rate. The curves were plotted with Origin™ 2018. Thermokinetical and thermodynamic parameters were calculated using Kissinger, Starink, KAS, FWO, KAS-Ir and FWO-Ir equations with Microsoft Excel.

### 2.3 Theoretical approach

The conversion of kaolin to metakaolin can be generally looked at as a one-step solid state reaction (eqn (1)).1
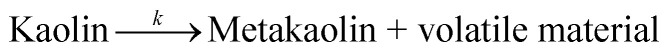
Here, *k* represents the rate constant (eqn (2)) and “volatile material” the water vapor from the dehydroxylation process.2
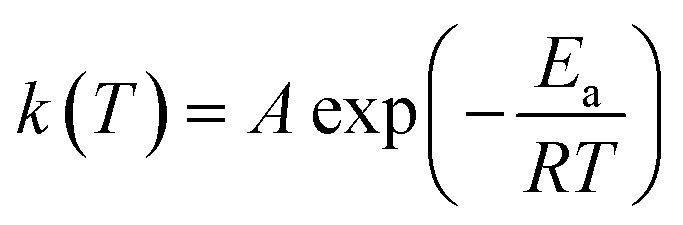


*E*_a_ represents the activation energy (kJ mol^−1^), *A* the pre-exponential factor (min^−1^), *T* the absolute temperature (*K*) and *R* the gas constant (J K^−1^ mol^−1^).

Non-isothermal kinetic analysis is a model-free method, in which measurements of the corresponding dehydroxylation temperatures *T* are functions of the degree of conversion *α* (eqn (3)) and a fixed value at different heating rates *β* (eqn (4)).3
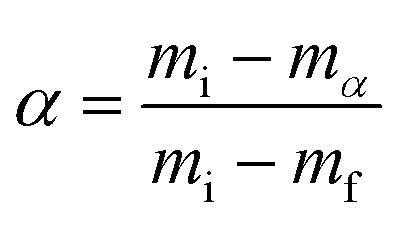
4
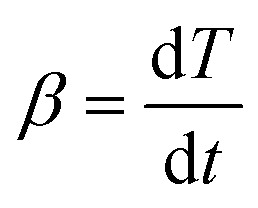
Here *m*_i_ is the initial mass of kaolin, *m*_*α*_ is the current mass at a certain degree of conversion *α* and *m*_f_ is the final mass. *t* is the time taken (min) and *β* the heating rate (K min^−1^).

Based on the kinetic theory, the kinetic equation of the thermal decomposition of solid-state (kaolin) materials is eqn (5).^37,38^5
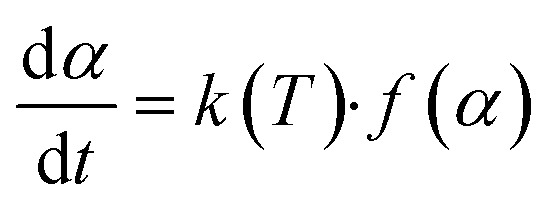
where *f*(*α*) is the reaction mechanism function of the dehydroxylation of kaolin.

Eqn (5) can be modified to eqn (6).6
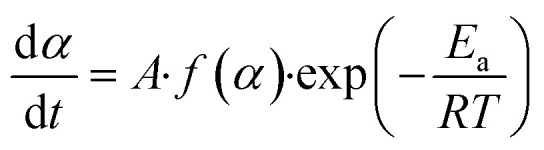


The substitution of eqn (4) within eqn (6) after adjustment, gives eqn (7), which is the fraction of material converted in a specified time.7
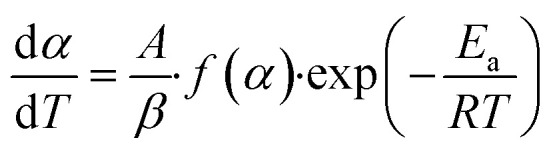


Eqn (7) can be solved by several methods. The solution of the integral form depends on the expression of the explicit function *f*(*α*) (Table S1)^39,40^ and its mechanism. The kinetic parameters of a solid state reaction are calculated based on the heating rates and linear fitting of TGA and DTG data as a function of the *α* and *T*.^29,40^ Transposition and integration of eqn (7) gives eqn (8).^41^8
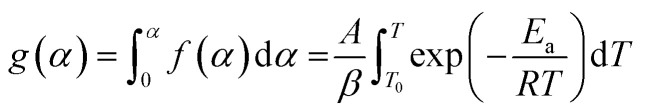
Here *g*(*α*) represents the integral form corresponding to each differential form of the function *f*(*α*). *T*_0_ and *T* are the initial and final pyrolysis temperatures for the reaction.

#### 2.3.1 Calculation of the activation energy *E*_a_

*E*_a_ was determined using integral methods based on KAS and FWO models, which consider DTG curves and correctly describe the calculation of kinetic parameters compared to those using differential methods (Kissinger and Ozawa). The differential method just considers the peak temperature *T*_m_, representing a process at a maximum conversion rate. According to integral methods, conversion is affected by *T* and *β*.^42,43^ These methods consider at least three values for *β* and the *g*(*α*) function represents the most probable mechanism function for the conversion.^29,40^ The Kissinger (eqn (9)) and Ozawa (eqn (10)) differential methods^44,45^ are given as follows:9
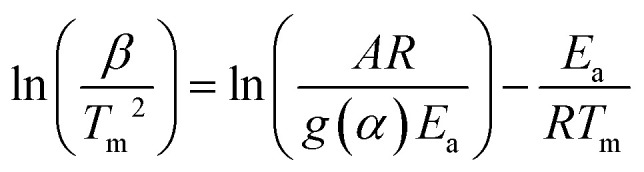
10
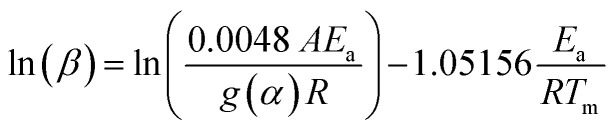
Here *T*_m_ is the maximum peak temperature of the DTG curve and *g*(*α*) the most probable mechanism function.

The integral methods of KAS given by eqn (11) (ref. 46) and FWO shown in eqn (12) (ref. 47 and 48) are as follows:11
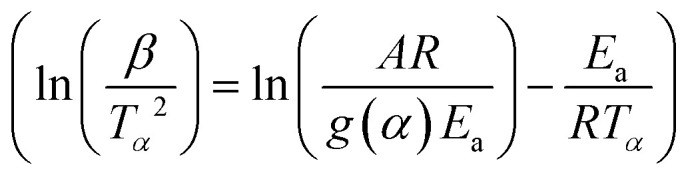
12
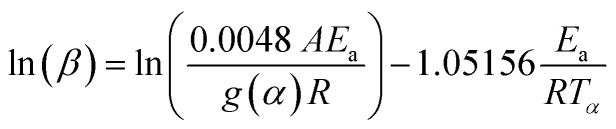


The *E*_a_ and *A* are obtained from the slope and intercept of the linear part of the model presenting the best *R*^2^ of the plot of ln(*β*/*T*_m_^2^) (Kissinger) and ln(*β*) (Ozawa) *versus* 1000/*T*_m_, for differential methods. Furthermore, these values can be obtained by plotting ln(*β*/*T*_*α*_^2^) (KAS) and ln(*β*) (FWO) *versus* 1000/*T*_*α*_ for the integral method, according to eqn (9)–(12), respectively.

The iterative procedure^39,40^ was applied to determine the most accurate value of *E*_a_ using the KAS-Ir (eqn (13)) and FWO-Ir (eqn (14)) methods.13
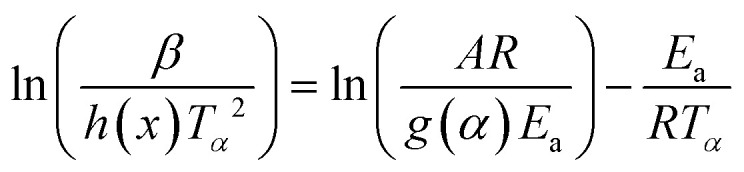
14
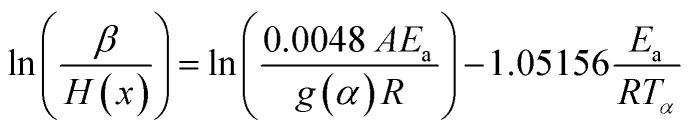
Here *h*(*x*) represents the 4th Senum and Yang approximation formula^49^ and *H*(*x*) is shown in eqn (16).15
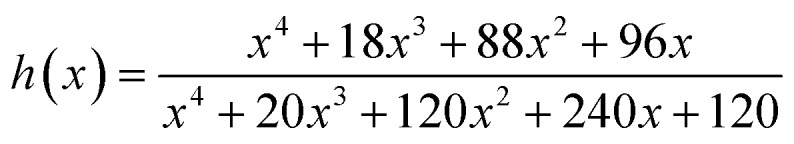
where *x* = *E*_a_/*RT*.16
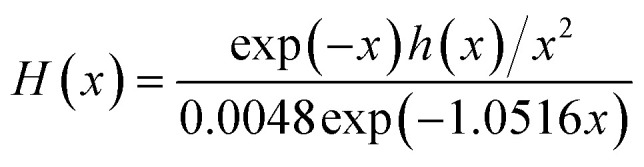


The iterative procedure^40,50^ followed this steps:

(i) It was assumed that *h*(*x*) or *H*(*x*) = 1. Then the initial value of *E*_a_(*E*_a1_) was calculated using KAS eqn (11) and FWO eqn (12). The model-free methods stop the calculation at this step.

(ii) The value of *E*_a1_ was used to calculate the value *E*_a2_ from the plot of ln[*β*/*h*(*x*)*T*_*α*_^2^] (KAS-Ir) or ln[*β*/*H*(*x*)] (FWO-Ir) *versus* 1000/*T*_*α*_ using eqn (13) and (14).

(iii) Step (ii) was then repeated after replacement of *E*_a1_ with *E*_a2_. This procedure was performed repeatedly until *E*_ai_ − *E*_a(i−1)_ < 0.01 KJ mol^−1^. Thus, the last value of *E*_ai_ was considered as the most accurate value of *E*_a_ of the reaction.

#### 2.3.2 Determination of the most probable mechanism function *g*(*α*)

The KAS and FWO models do not consider *g*(*α*). Therefore, it was determined using two other methods: the MHR method^51^ and the Coats-Redfern method. The Coats-Redfern model is recommended by the International Confederation for Thermal Analysis and Calorimetric (ICTAC) Kinetics Committee.^52^

For the MHR method eqn (17) was used in its linear form, derived by the integration of the right-hand side of eqn (8) giving eqn (17) with *x* = *E*_a_/*RT*.17
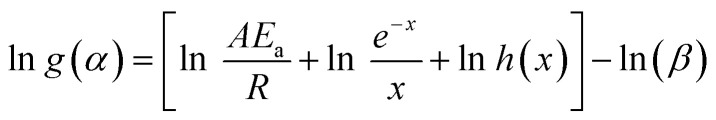


Linear regression of the plot of ln *g*(*α*) *versus* ln(*β*) provides an information on the probability of *g*(*α*) to represent the reactions mechanism. The closer the linear coefficient of correlation *R*^2^ is to 1 and the closer the slope is to −1, the more likely the tested function is describing the reaction mechanism. Eqn (17) shows that *A* and *E*_a_ have no effect on *g*(*α*). Thirty-six functions (Table S1) were tested with the MHR method to obtain the most probable function, where different values of conversion *α* corresponding to each heating rate *β* taken at the same temperature value were implemented in eqn (17).^39,53^

For comparison we use the Coats-Redfern model (eqn (18)), which can be adjusted using a single heating rate.18
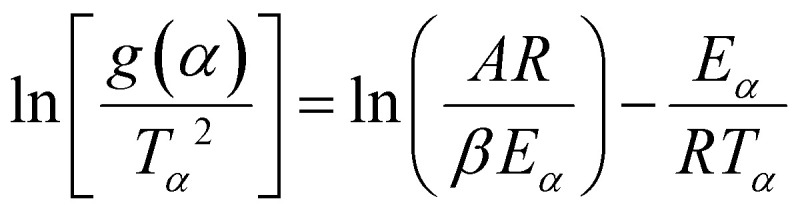


The same 36 functions as for the MHR method were tested. When the correlation coefficient *R*^2^ of the plot of ln[*g*(*α*)/*T*_*α*_^2^] *versus* 1000/*T*_*α*_ for one function is the closest to 1 this function is assumed the most probable mechanism function.

#### 2.3.3 Calculation of the pre-exponential factor *A*

*A* was determined using the intercepts of the plots of the KAS-Ir and FWO-Ir models (eqn (13) and (14)), after inserting the values of *E*_a_ corresponding to each conversion rate *α*, the gas constant *R* and the beforehand determined most probable *g*(*α*).

#### 2.3.4 Determination of the thermodynamic parameters Δ*S*^≠^, Δ*H*^≠^ and Δ*G*^≠^ of the kaolin conversion

The relationship between the thermodynamic parameters of the conversion process and the kinetic parameters is a function of the correlation between the Zenera and Wertera or Eyring and Arrhenius laws, related to the reactions rate constant *k*(*T*).^35,54^ Δ*S*^≠^, Δ*H*^≠^ and Δ*G*^≠^ were calculated using eqn (19)–(21), respectively.19
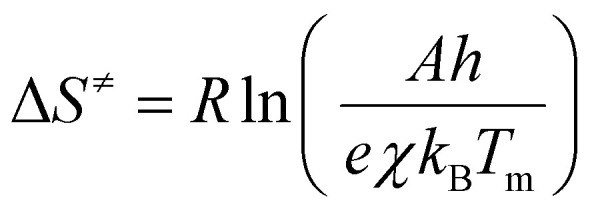

20 Δ*H*^≠^ = *E*_*α*_ − *RT*_m_

21 Δ*G*^≠^ = Δ*H*^≠^ − *T*_m_Δ*S*^≠^

Herein *E*_*α*_ is obtained from the iterative procedure using the KAS-Ir and FWO-Ir models, *T*_m_ is the peak temperature of the DTG curve, *A* is the pre-exponential factor, *h* is Planck's constant (*h* = 6.6269 · 10^−34^ J s), *e* is the Neper number (*e* = 2.7183), *χ* is the transition factor equal to unity,^37,55^*k*_B_ is the Boltzmann constant (*k*_B_ = 1.3819 · 10^−23^ J K^−1^) and *R* is the gas constant (*R* = 8.314 J mol^−1^ K^−1^).

### 2.4 Characterization techniques

The identity of all samples was determined by XRD using an Empyrean diffractometer (PANanalytical, Almelo, The Netherlands) equipped with a Cu-tube (*λ*: *K*_*α*1_ = 1.540598 Å and *K*_*α*2_ = 1.544426 Å) operating at 40 mA and 40 kV. All samples were scanned from 4–90° 2*θ*. The chemical composition of the raw kaolin was quantified using a PANanalytical Cubix-2300 XRF spectrometer. The morphology and microstructure of all samples was recorded with an SEM XL30 FE SEM (FEI, Hillsboro, USA) at an acceleration voltage of 20 kV. FT-IR data was recorded using a Nicolet iS50 IR spectrometer (Thermo Scientific, Schwerte, Germany) in the 4000–200 cm^−1^ wavenumber range. TGA and DTA were performed using a TGA-701 (LECO, St. Joseph, USA) for large samples (∼1.5 g) and an STA 449C Jupiter (Netzsch, Selb, Germany) thermogravimetric analyzer for small sample (∼20 mg).

## 3 Results and discussion

### 3.1 X-ray fluorescence (XRF) analysis

The composition of kaolin was determined using XRF analysis (Table 1). The kaolin used for this work consists mainly of silicon oxide (52.02%) and aluminum oxide (34.01%) as well as minor impurity phases such as iron oxide (0.55%) and titanium oxide (1.20%). The heat treatment leads to the conversion of kaolin into an amorphous and highly reactive phase, which enhances the nucleation kinetics and rearrangement of silica-alumina oligomers during the zeolitization step.^56,57^ A high content of metal oxides and quartz present in the kaolin starting material has a negative impact on the zeolite yield.^58^

### 3.2 X-ray diffraction analyses of kaolin and its products from thermal treatment MK-(5), MK-(20) and MK-(40)

**Fig. 1:**
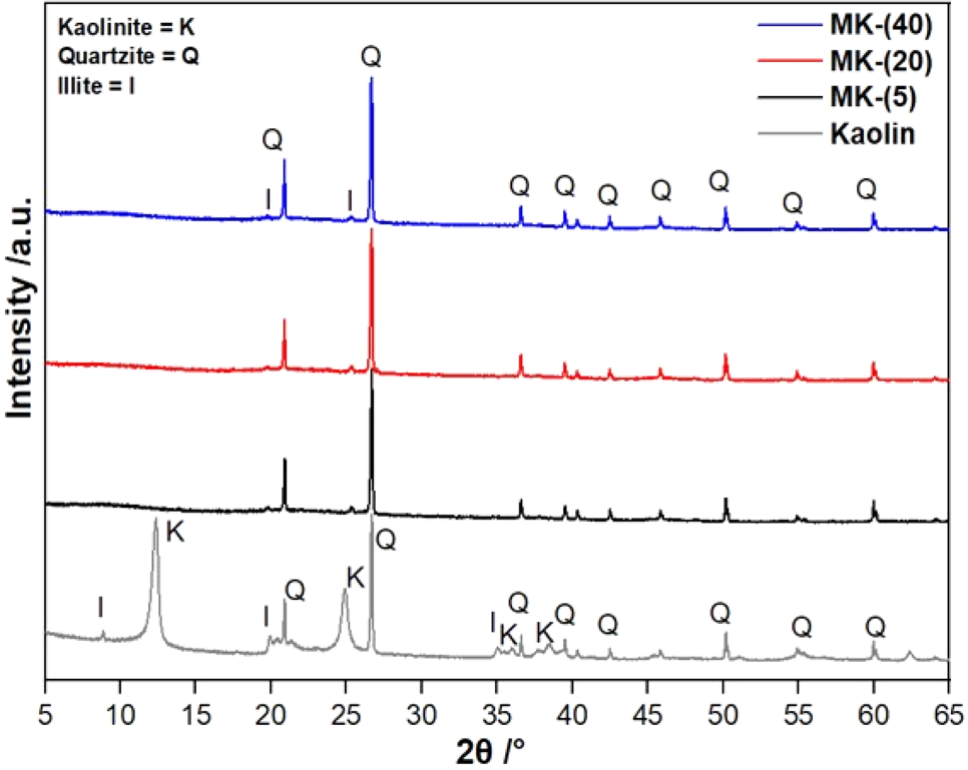
XRD patterns of cameroonian kaolin and derived metakaolin samples MK-(5), MK-(20) and MK-(40), achieved by TGA measurements using *β =* 5, 20 and 40 K min^−1^. The kaolin pattern shows the presence of kaolinite (*K*), illite (*I*), quartz (*Q*) and an amorphous phase. The measurements were performed using a PANalytical Empyrean diffractometer with Cu *K*_*α*1+*α*2_ radiation.

Fig. 1 shows the XRD patterns of cameroonian kaolin and its calcination products. The pattern of kaolin shows mostly kaolinite^59^ together with illite and quartz.^60^ These phases were identified by comparison with standard data from the JCPDS database.^61^ The samples derived from the thermal activation procedures were lacking the characteristic peaks of kaolinite, but show a higher amorphous background (Fig. S2) and remaining reflections of quartz and small amounts of illite. We assume the alteration of the ordered crystalline structure of kaolinite to an amorphous phase by elimination of the hydroxyl groups present on the surface of the octahedrally coordinated aluminum layer with temperatures above 800–1000 °C, which is typical for this 1 : 1 (T : O = tetrahedral/octahedral) clay mineral.^62^ The dominant crystalline phase in the obtained metakaolin samples is quartz.^60^ Furthermore, samples activated at different heating rates showed the same phases. From XRD it is not easy to determine the degree of conversion of the kaolin as the quartz phase is highly crystalline and dominating the diffraction pattern of the products and metakaolin is an amorphous phase. Furthermore, the formation of metakaolin is visible by strong delamination at around 800–950 °C.^35^ At around 990 °C, crystalline phases form as a result of the gradual oxidation of metakaolin into silicon-aluminum spinel.^59^ Above 990 °C, the spinel phase decomposes and transforms into mullite and cristobalite phase.^30^ The degree of conversion is better visible in the SEM data (chapter 4.1).

### 3.3 SEM analysis of cameroonian kaolin and its products from thermal treatment MK-(5), MK-(20) and MK-(40)

Fig. 3 shows the FE-SEM images of kaolin and metakaolin. The figure displays a typical, lamellar morphology of kaolinite (top left), with a parallel layer stacking.^63^ The layers show some discontinuities and variable thicknesses, which can be explained by the presence of impurities in the kaolin material.^6–8^ The metakaolin MK-(5) (top right) presents a morphology with altered parallel layers and more disorder, indicating that the stacking of the tetrahedral layers has been destroyed. The morphological change of metakaolin resulting from the treatment at 5 K min^−1^ is more significant compared to the samples MK-(20) and MK-(40) (bottom left and right). These results agree with the results from Irfan Khan *et al.* 2017,^29^ who reported, that the dehydroxylation of kaolin starts from the external Al–OH groups and progresses towards the internal groups. We assume, as lower heating rates delivered more time to reach the final temperature, which enhanced the dehydroxylation of most of the external Al–OH and Si–OH bonds,^66^ the layer stripping was intensified. This means that low heating rates enhance the dehydroxylation of kaolin layers and the formation of more disordered structures.^33^

### 3.4 FT-IR analyses of cameroonian kaolin and its products from thermal treatment MK-(5), MK-(20) and MK-(40)

**Fig. 2:**
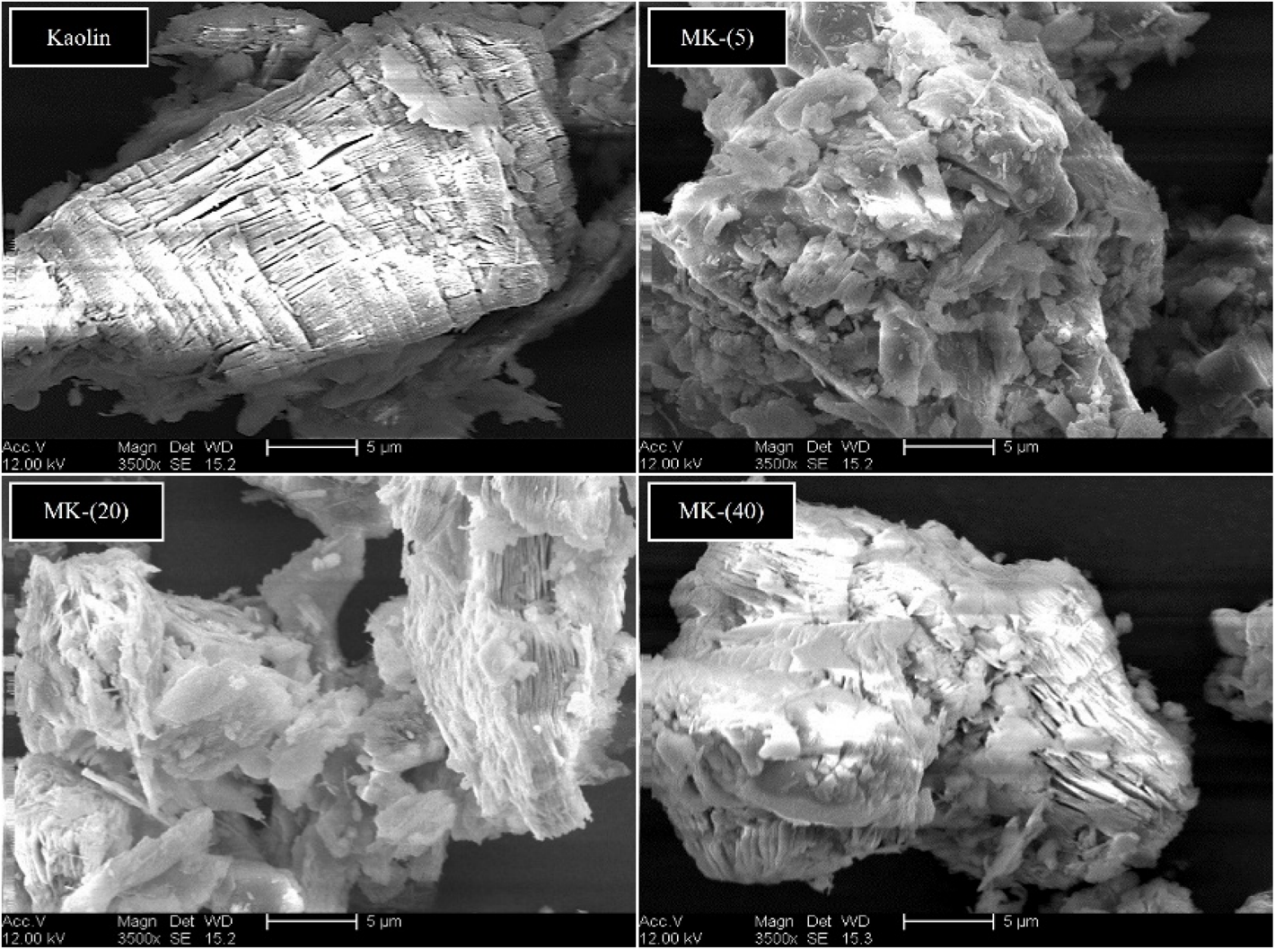
SEM images of kaolin (top left) and metakaolin samples MK-(5) (top right), MK-(20) (bottom left) and MK-(40) (bottom right), achieved by TGA measurements using *β =* 5, 20 and 40 K min^−1^.

**Fig. 3:**
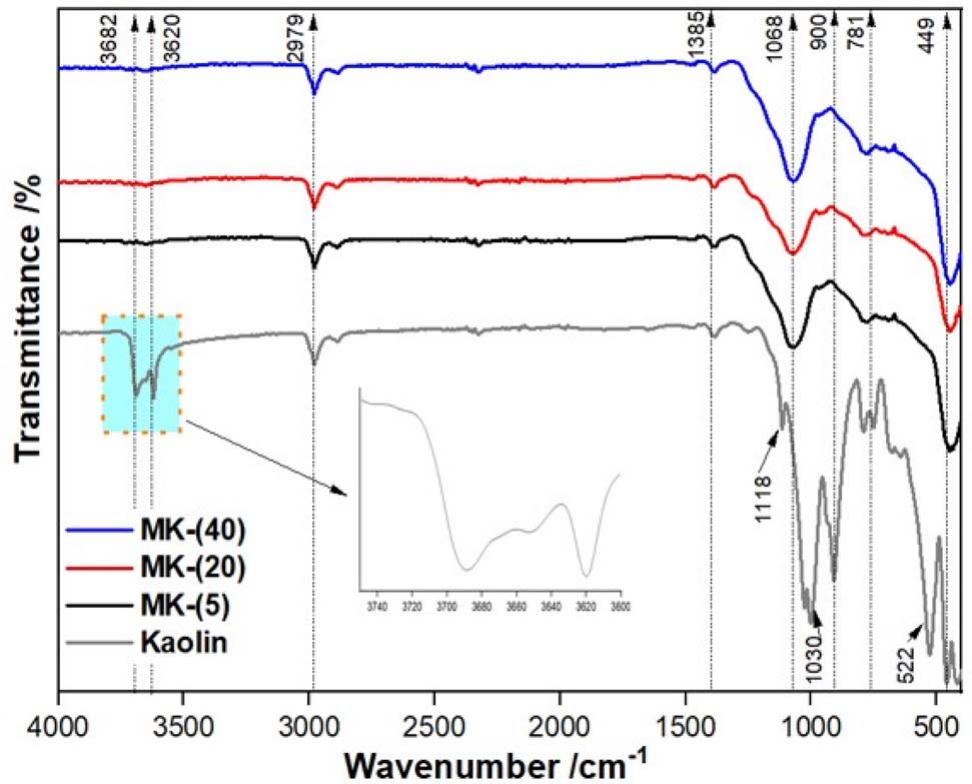
FT-IR spectra of cameroonian kaolin and metakaolin samples MK-(5), MK-(20) and MK-(40), achieved by TGA measurements using *β =* 5, 20 and 40 K min^−1^.

Fig. 3 displays the FT-IR spectra of cameroonian kaolin and the metakaolin samples. The strong bands at 3690 and 3650 cm^−1^ present in the FT-IR spectrum of the kaolin sample are attributed to O–H stretching vibrations of interlayer hydroxyl groups in kaolinite.^29^ Additionally, the broad band around 3620 cm^−1^ is attributed to the O–H bonds of water molecules bound to kaolinites tetrahedral layers.^64^ The bands around 2979–2900 cm^−1^ are assigned to Si–OH elongation vibration bands. The bands at 1385, 1118 and 1030 cm^−1^ correspond to the asymmetric stretching vibrational bands of Al–O and Si–O bonds.^67^ Similarly, the bands at 900, 781, 522 cm^−1^ are characteristic for the vibrations of Al–O, Si–O–Si and Al–O–Si in the natural structure of kaolin.^60^ The disappearance of the strong bands at 3690, 3650 and 3620 cm^−1^ in the FT-IR spectra of the metakaolin samples indicates the transformation of kaolin to metakaolin by a dehydroxylation process.^68^ The absorption bands around 2979–2900 and 1385 cm^−1^, visible in all samples, show the presence of antisymmetric Si–O and Al–O vibrations from tetrahedrally coordinated silicon and aluminum atoms. The formation of metakaolin is also confirmed by the presence of new vibration bands (Fig. 2, spectra from MK-(5), MK-(20) and MK-(40)) at 1068 and 449 cm^−1^. These bands can be attributed to the Al–O–Si and Si–O–Al bonds in amorphous metakaolin (Fig. 3).^13,14^

## 4 Thermal analysis and kinetics of the cameroonian kaolin conversion process

### 4.1 TGA, DTG and DTA

**Fig. 4:**
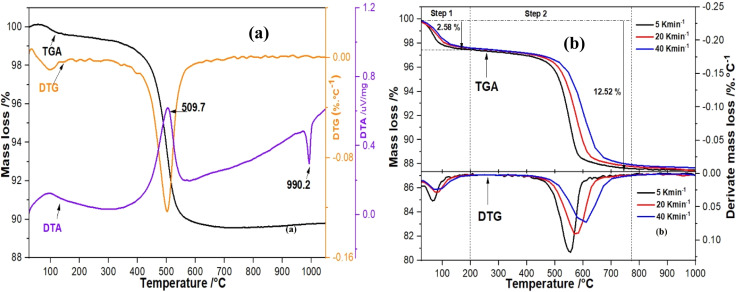
(a) TGA/DTG/DTA data of cameroonian kaolin collected using an STA 449C Jupiter (Netzsch, Selb, Germany) with 5 K min^−1^; (b) TGA/DTG data of cameroonian kaolin collected using a TGA-701 (LECO, St. Joseph, USA) with *β =* 5, 20 and 40 K min^−1^.

Fig. 4(a) displays the TGA/DTG/DTA curves of cameroonian kaolin collected using *β =* 5 K min^−1^.^33^ It shows two endothermic peaks in the DTA curve and the associated mass losses in the TGA curve. The step between 30–200 °C, represents the removal of moisture and adsorbed water molecules^30^ (denoted step 1). The step between 400–600 °C arises from the destruction of the layers and the dehydroxylation of the kaolinite structure (denoted step 2). The data is typical for kaolin as previously reported.^59,60^ The behavior is explained by the high content of kaolinite in the cameroonian kaolin, which contributes to the formation of highly reactive metakaolin with tetrahedrally coordinated aluminum.^72^ Furthermore, the DTG curve shows a maximum mass loss at ∼555 °C. This results from endothermic dehydroxylation and the recombination of silicon oxide and aluminum oxide building units^73–75^ leading to the formation of disordered metakaolin, where the coordination number (C.N.) of silicon in the tetrahedral layers remains 4 and the C.N. of aluminum in the octahedral layers changes from 6 to 4.^27,35^ The third exothermic peak at around 990 °C arises from the formation of crystalline phases, due to the gradual oxidation of metakaolin to silicon–aluminum spinel.^59^ The spinel phase decomposes above 990 °C and changes to highly crystalline cristobalite and mullite.^30^

Fig. 4(b), shows the simultaneous TGA and DTG analyses of the kaolin dehydroxylation using *β =* 5, 20 and 40 K min^−1^. The figure illustrates a shift of the DTG peaks for step 1 and step 2 corresponding to *β*, characterized by the variation of the medium temperature *T*_m_ which was 72.9, 83.3 and 95.2 °C for step 1 and 555.9, 584.2 and 611.7 °C for step 2, respectively. This observation can be explained by the lower total amount of energy supplied to the sample due to higher *β*.^11^ The shift of *T*_m_ can be attributed to the deformation of Si–O–Al bonds between the most external tetrahedral and octahedral kaolin layers and those located towards the crystal core.^13,63^ The shift in the DTG peak minima related to the medium temperature is the basis for the kinetic study applying FWO, KAS, Kissinger and Starink models. Similar variations were observed by Irfan Khan *et al.*, 2017.^29^ In addition, the significant overlap of the TGA curves between 300–450 °C shows the general onset of the conversion process.^34^

Recently, Ptáček *et al.*, 2011, reported that the peak height of the DTG curves decreased with increasing *β*, which could justify a partial conversion of kaolin to metakaolin. Therefore, a lower *β* would be more beneficial for the production of high-quality metakaolin. Furthermore, the formation of metakaolin is visible by strong delamination at around 800–950 °C.^35^ At around 990 °C, crystalline phases form as a result of the gradual oxidation of metakaolin into silicon–aluminum spinel.^59^ Above 990 °C, the spinel phase decomposes and transforms into highly crystalline cristobalite and mullite. Additionally, amorphous silicas crystallize into cristobalite.^30^ Indeed, slightly decreasing mass losses of 12.52, 12.12 and 12.01 weight% were observed for samples treated at 5, 20 and 40 K min^−1^, respectively. Step 2 showed a mass loss of approximately 10.501%, 9.98%, and 9.01% for samples treated at 5, 20 and 40 K min^−1^, respectively which confirmed the downward trend in total mass loss. The variation in weight loss illustrates the impact of *β* during the heat treatment of kaolin.^64,65^ An additional justification for the incomplete transformation at higher *β* is the calcination time, which remained insufficient for the water molecules in the core of the particles to leave by diffusion.^30^

The dehydroxylation degrees D_d_ of 0.958 at 555 °C and 0.998 at 785 °C (applying eqn S1 on the TG curve from 5 K min^−1^) also fit to the mass loss during metakaolinization. It results from the decomposition of the hydroxyl groups bound to the octahedral and tetrahedral sheets.^1,30^ Recently, Bich *et al.*, 2009,^68^ reported that metakaolin with D_d_ above 0.950 shows an increased reactivity.

### 4.2 Thermokinetic study

#### 4.2.1 Determination of *E*_a_ of the conversion of cameroonian kaolin to metakaolin

The differential methods of Kissinger and Ozawa and the integral methods of KAS and FWO were applied based on the maximum peak temperature of the DTG curves and TGA data. Iterative procedures KAS-Ir and FWO-Ir were employed to determine the accurate *E*_a_.

##### 4.2.1.1 *E*_a_ according to the Kissinger and Ozawa methods

*E*_a_ for step 1 and step 2 was determined from the slope of the plot of ln(*β*/*T*_m_^2^) and ln(*β*) *vs.* 1000/*T*_m_ for Kissinger and Ozawa methods, respectively (Fig. S3a and b). The values of *E*_a_ and *A* determined using the DTG peak data are presented in Table S2. *E*_a_ was 73.089 and 93.614 kJ mol^−1^ for step 1 and 254.515 and 261.839 kJ mol^−1^ for step 2 for Kissinger and Ozawa methods, respectively. Both methods showed a significant difference in the values of *E*_a_ and *A* for step 2 as for step1. The difference between both methods for step 1 could be due to the variation of the maximum peak temperature and *β*. Kissinger's method considers *β* unlike Ozawa's method.^29^ Furthermore, Kissinger's method was less accurate than Ozawa's, as shown by the lower *R*^2^ values obtained (Table S2). Recently, Zemenová *et al.*, 2014, showed that Kissinger's equation was not suitable to determine *E*_a_ of the first step of kaolin conversion. Moreover, it is noticed that the *R*^2^ values are smaller for step 1 compared to step 2, which might be due to the lower temperature values at step 1 (Table S2). Nevertheless, the *E*_a_ values for step 2 are close to those from literature.^34^

##### 4.2.1.2 *E*_a_ using the iterative procedure following the KAS-Ir and FWO-Ir methods

The first values of *E*_a_ for steps 1 and 2 were determined from the slope of the plot of ln(*β*/*T*_*α*_^2^) *vs.* 1000/*T*_*α*_ for KAS (Fig. S4a and b) and ln(*β*) *vs.* 1000/*T*_*α*_ for FWO models (Fig. S5a and b). The first values of *E*_a_ for *α* from 0.1 to 0.9 were between 56–91 KJ mol^−1^ and 93–105 KJ mol^−1^ for step 1 and 231–280 kJ mol^−1^ and 239–286 kJ mol^−1^ for step 2 using the KAS and FWO models, respectively (Table S3). The average values of the derived energies for each model were 77.27 kJ mol^−1^ and 97.61 kJ mol^−1^ for step 1 and 252.15 kJ mol^−1^ and 259.39 kJ mol^−1^ for step 2. The gap between the values from the two models is >5%, indicating a low reliability of the first values of *E*_a_, due to the integral approximation of the conversion temperatures.^76^ Remarkably, *E*_a_ obtained using the FWO model was higher compared to the KAS model. This difference is driven by high *β* and the low temperature. It shows that the conversion process is governed by several reaction steps.^77^ Chen *et al.*, 2012,^36^ reported that multi-step mechanisms are dominated by a significant variation of *E*_a_. It is recommended that the KAS and the FWO method should only be used including the iterative procedure.^78–80^

**Fig. 5:**
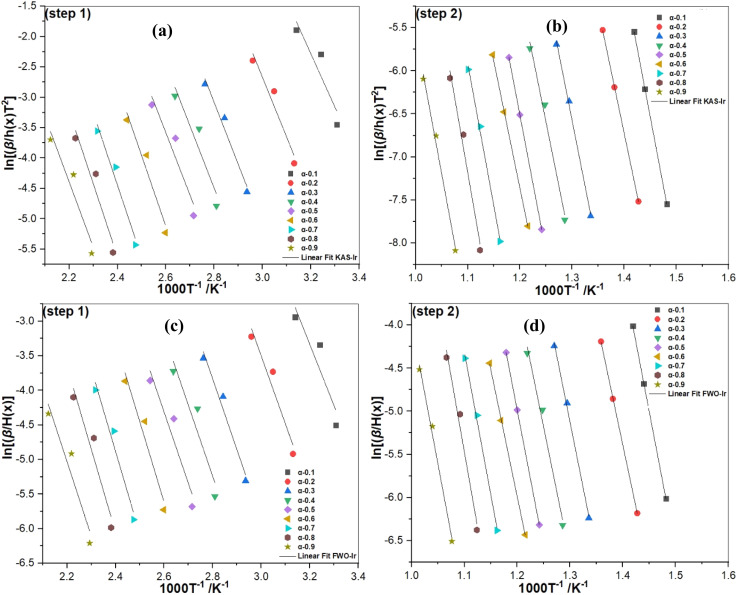
Thermokinetics parameter plots for the conversion of kaolin using KAS-Ir (a and b) and FWO-Ir (c and d) methods for step 1 and 2 based on DTG data together with the iterative procedure.

**Fig. 6:**
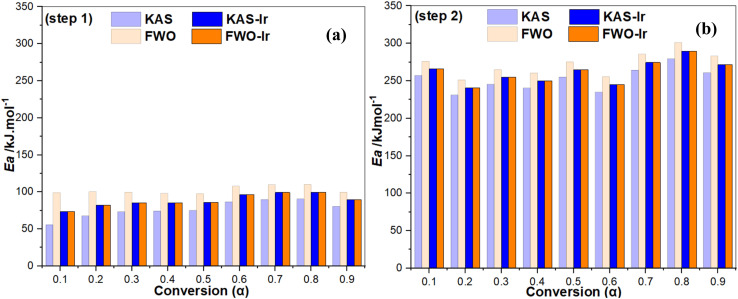
*E*_a_ *versus* degree of conversion *α* for dehydration (step 1) (a) and dehydroxylation (step 2) (b) during the cameroonian kaolin conversion, calculated following the KAS and the FWO method. KAS-Ir and FWO-Ir describe the values derived from the iterative procedure.

*E*_a_ and *R*^2^ obtained by applying the iterative procedure of KAS-Ir (Fig. 5a and b) and FWO-Ir (Fig. 5c and d) for dehydration and dehydroxylation were calculated using the eqn (13) and (14) (Table 2). The *E*_a_ values range from 73.32–99.69 kJ mol^−1^ and 73.71–99.75 kJ mol^−1^ for step 1 and from 240.69–289.73 kJ mol^−1^ and 240.75–289.79 kJ mol^−1^ for step 2, for the KAS-Ir and FWO-Ir models, respectively. The average global *E*_a_ were 88.44 and 88.58 kJ mol^−1^ for step 1 and 261.85 and 261.91 kJ mol^−1^ for step 2. The obtained values are close to those reported by Irfan Khan *et al.*, 2017.^29^ The linear correlation coefficients *R*^2^ (Table 2) of the KAS-Ir model are closer to 1 than those of the FWO-Ir model. Nevertheless, the *E*_a_ values obtained by the iterative procedure of KAS-Ir and FWO-Ir are very close to each other (Δ ≈ 0.1 kJ mol^−1^) in contrast to the results without the iterative procedure (Δ ≈ 2.52–9.03 kJ mol^−1^, Table S3). This indicates that the FWO model's approximation was less accurate than the KAS model's. The variation of activation energies allows to conclude that the 4th Senum and Yang approximation^49^ is suitable to evaluate the accurate values of *E*_a_. Therefore, the FWO and KAS methods should always be used with iterative correction.^67^ The iterative method is more reliable, significantly improving regression coefficients and energy accuracy. Additionally, it was observed that *E*_a_ values depend on the degree of conversion, suggesting that the delamination process is controlled by one-step reactions. Furthermore, obviously *E*_a_ is dependent on *α* (Fig. 6a and b). Within step 2, this observation indicates that the delamination and dehydroxylation process is controlled by a single step reaction.^68^ In addition, the variation of *E*_a_ as a function of *α* shows the nonconformity of the hydroxyl groups bound on the tetrahedral and octahedral layers of kaolin. The higher *E*_a_ values can be assigned to the dehydroxylation of the hardly accessible –OH groups.^59^ Furthermore, the increase in *E*_a_ beyond *α* = 0.5 shows that the dehydroxylation process is complex and involves several chemical mechanisms.^30^

**Table 2:** *E*_a_, *A* and *R*^2^ calculated using the iterative procedure of KAS-Ir and FWO-Ir methods for step 1 and 2

	*α*	Step 1			Step 2		
		*E*_a_/kJ mol^−1^	*R* ^2^	*A*/min^−1^	*E*_a_/kJ mol^−1^	*R* ^2^	*A*/min^−1^
KAS	0.1	73.32	0.874	3.18 × 10^9^	265.97	0.999	8.87 × 10^18^
	0.2	81.81	0.939	1.74 × 10^11^	240.69	0.999	3.49 × 10^16^
	0.3	85.07	0.970	2.86 × 10^11^	254.69	0.997	3.94 × 10^16^
	0.4	85.17	0.908	2.01 × 10^11^	249.89	0.986	5.88 × 10^15^
	0.5	85.60	0.911	1.62 × 10^11^	264.60	0.986	1.94 × 10^16^
	0.6	96.47	0.946	2.71 × 10^12^	244.88	0.999	6.06 × 10^14^
	0.7	99.22	0.961	2.36 × 10^12^	274.53	0.999	1.28 × 10^16^
	0.8	99.69	0.932	1.59 × 10^12^	289.73	0.982	5.05 × 10^16^
	0.9	89.58	0.920	5.87 × 10^10^	271.70	0.994	1.69 × 10^15^
**Average**		**88.44**		**8.39 × 10** ^**11**^	**261.85**		**1.01 × 10** ^**18**^
FWO	0.1	73.71	0.884	1.53 × 10^12^	266.04	0.999	8.58 × 10^18^
	0.2	82.05	0.939	1.17 × 10^12^	240.75	0.999	5.02 × 10^16^
	0.3	85.22	0.970	1.95 × 10^11^	254.75	0.997	4.76 × 10^16^
	0.4	85.28	0.902	5.82 × 10^10^	249.95	0.986	7.82 × 10^15^
	0.5	85.71	0.911	2.96 × 10^10^	264.66	0.986	2.42 × 10^16^
	0.6	96.56	0.946	2.64 × 10^11^	244.94	0.999	9.16 × 10^14^
	0.7	99.29	0.961	1.58 × 10^11^	274.58	0.996	1.66 × 10^16^
	0.8	99.75	0.932	8.91 × 10^10^	289.79	0.982	6.15 × 10^16^
	0.9	89.63	0.920	3.54 × 10^9^	271.75	0.994	2.54 × 10^15^
**Average**		**88.58**		**3.89 × 10** ^**11**^	**261.91**		**9.78 × 10** ^**17**^

#### 4.2.2 Determination of the most probable mechanism function *g*(*α*) of the conversion of cameroonian kaolin to metakaolin

**Fig. 7:**
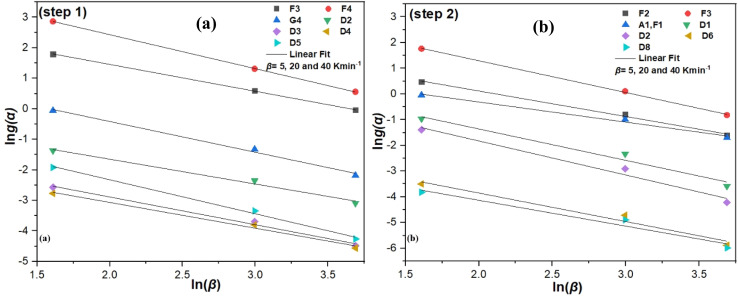
Plot of ln *g*(*α*) *versus* ln(*β*) for step 1 (a) and step 2 (b) of the kaolin conversion, referring to the three experiments at *β* = 5, 20, 40 K min^−1^.

The MHR method (eqn (17)), was used to find *g*(*α*) for the dehydration and dehydroxylation of kaolin. Two suitable temperatures (*T*_1_ = 72.908 °C and *T*_2_ = 555.884 °C) were selected for the three experiments with *β* = 5, 20 and 40 K min^−1^ and the respective *α* were calculated (Table S4). The most probable *g*(*α*) was determined by plotting ln *g*(*α*) *vs.* ln(*β*) for steps 1 and 2, respectively. For these two steps 36 functions (Table S1) were tested. The most probable *g*(*α*) chosen was the one with a slope close to −1 and the linear regression coefficients *R*^2^ close to 1 (Fig. 7a and b; Table 3). The models F_3_, F_4_, G_4_, D_2_, D_3_, D_4_ and D_5_ for step 1 and the models F_2_, F_3_, D_1_, A_1_, F_1_, D_2_, D_6_ and D_8_ for step 2 resulted in the best *R*^2^ values. For step 1 the model G_4_ showed the closest slope to −1. For step 2 the model F_2_ showed the closest slope to −1. Furthermore, although the *R*^2^ of G_4_ was not more accurate than those of F_3_ and F_4_, G_4_ showed the best slope. This indicates the models G_4_ and F_2_ as the most probable *g*(*α*). The results show that the dehydration of kaolin is affected by the random nucleation and subsequent growth mechanisms (G_4_) and the dehydroxylation of kaolin is affected by a chemical reaction of second order (F_2_). Furthermore, these functions reveal the stability of the activated complex during the dehydroxylation process. Additionally, these functions suggest that the spontaneous dehydroxylation of –OH groups may not occur at low temperatures, indicating that the second stage of decomposition is more difficult than the first.

**Table 3:** Symbols of the algebraic expressions (detailed expression in Table S1) of the function *g*(*α*), *R*^2^ and slopes obtained using the MHR method for the investigation of cameroonian kaolin dehydroxylation

Step	Symbol for *g*(*α*)	*R* ^2^	Slope
1	F3	0.99981	−0.87916
	F4	0.99995	−1.11142
	G4	0.99377	−1.00586
	D2	0.98806	−0.81150
	D3	0.99133	−0.90650
	D4	0.98928	−0.84301
	D5	0.99568	−1.11289
2	F2	0.99571	−0.99111
	F3	0.99921	−1.23539
	D1	0.97306	−1.22006
	A1, F1	0.98765	−0.88210
	D2	0.97847	−1.32307
	D6	0.96822	−1.10844
	D8	0.96264	−1.00432

**Fig. 8:**
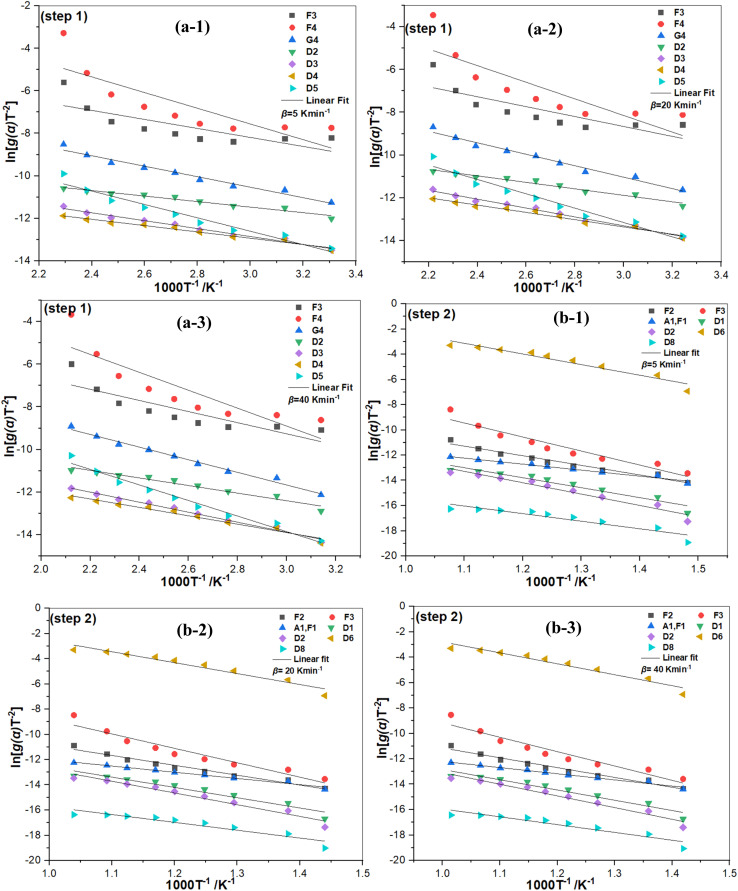
Plots following the Coats-Redfern model to determine the thermokinetics of the kaolin conversion to metakaolin for step 1 (a) and step 2 (b) at heating rate of 5 K min^−1^ (a-1 and b-1), 20 K min^−1^ (a-2 and b-2) and 40 K min^−1^ (a-3 and b-3).

In parallel, the Coats-Redfern model (eqn (18)) was used to confirm the most probable *g*(*α*), using the same 36 functions (Table S1). The best suited *g*(*α*) was determined by plotting ln[*g*(*α*)/*T*_*α*_^2^] *vs.* 1000/*T*_*α*_ for step 1 (Fig. S6(a-1, a-2 and a-3)) and step 2 (Fig. 8(b-1, b-2 and b-3)). The different functions with the best *R*^2^ values are shown in Table S5. The models G_4_ and F_2_ were selected as the most probable functions for steps 1 and 2, consistently with the results of the MHR method. The overall results show that the dehydration and the dehydroxylation of kaolin are affected by the random nucleation and subsequent growth mechanisms (G_4_) and a chemical reaction of second order (F_2_). The integral form of these functions are *g*(*α*) = [−ln(1 − *α*)]^2^ and *g*(*α*) = (1 − *α*)^−1^ – 1, for G_4_ and F_2_, respectively. Similar results have been reported by,^30,69–71^ which underlines the reliability and plausibility of the obtained *g*(*α*) in our study.

#### 4.2.3 Evaluation of the pre-exponential factor (*A*) of the conversion of cameroonian kaolin to metakaolin

*A* was calculated from the intercepts of the plots of eqn (13) and eqn (14) for KAS-Ir and FWO-Ir models (Fig. 5), inserting *E*_a_ obtained from the iterative procedure corresponding to each degree of conversion (*α*) and the most probable *g*(*α*).^81^ The average value of *A* (Table 2) for step 1 is 8.39 × 10^11^ min^−1^ (for KAS-Ir) and 3.89 × 10^11^ min^−1^ (for FWO-Ir) and for step 2 it is 1.01 × 10^18^ min^−1^ (for KAS-Ir) and 0.97 × 10^18^ min^−1^ (for FWO-Ir). The values of *A* increased reciprocally with temperature. This could be explained by the presence of complex reactions during the dehydroxylation.^82^ In addition, the values of *A* showed a gradual reduction with the increase of *α*. Furthermore, the large values of *A* for *α* < 0.5 suggest that the conversion reaction was more complex. The obtained small values of *A* for *α* > 0.5 suggest a low reactivity, which implies that the dehydroxylation of kaolin was spontaneous at high temperatures.^35^

### 4.3 Determination of the thermodynamic variables (Δ*S*^≠^, Δ*H*^≠^, Δ*G*^≠^) of the conversion of cameroonian kaolin to metakaolin

Δ*S*^≠^, Δ*H*^≠^, Δ*G*^≠^ of the kaolin conversion are summarized in Table 4. For step 1 the KAS-Ir and FWO-Ir models resulted in Δ*S*^≠^ < 0, *i.e.* −36.41 J mol^−1^ K^−1^ and −41.91 J mol^−1^ K^−1^, respectively. This could be explained by the formation of an activated complex during the dehydration of kaolin with a more ordered transition state. For step 2 both models resulted in Δ*S*^≠^ > 0, *i.e.* 55.88 J mol^−1^ K^−1^ and 53.00 J mol^−1^ K^−1^, respectively. These values are related to the dehydroxylation of kaolin and the formation of metakaolin. They might be explained by the formation of the highly disordered structure of metakaolin derived by a structural rearrangement of the activated complex during the delamination step.^37^

**Table 4:** Thermodynamic parameters (Δ*S*^≠^/J mol^−1^ K^−1^, Δ*H*^≠^/kJ mol^−1^ and Δ*G*^≠^/kJ mol^−1^) for the conversion of cameroonian kaolin to metakaolin

*α*	KAS-Ir			FWO-Ir		
	Δ*S*^≠^	Δ*H*^≠^	Δ*G*^≠^	Δ*S*^≠^	Δ*H*^≠^	Δ*G*^≠^
Step 1						
0.1	−72.50	70.45	95.42	−21.13	70.85	78.13
0.2	−39.23	78.95	92.45	−23.39	79.18	87.24
0.3	−35.10	82.21	94.29	−38.24	82.36	95.52
0.4	−38.05	82.30	95.41	−48.33	82.42	99.06
0.5	−39.82	82.74	96.45	−53.94	82.84	101.44
0.6	−16.40	93.61	99.26	−35.77	93.70	106.00
0.7	−17.54	96.36	102.40	−40.01	96.43	110.24
0.8	−20.79	96.82	103.97	−44.80	96.89	112.32
0.9	−48.26	86.71	103.35	−71.60	86.77	111.42
**Avg**	**−36.41**	**85.57**	**98.11**	**−41.91**	**85.72**	**100.15**

Step 2						
0.1	101.01	259.08	175.34	100.73	259.15	175.63
0.2	54.97	233.80	188.22	57.98	233.86	185.78
0.3	55.96	247.80	201.40	57.54	247.86	200.15
0.4	40.15	243.00	209.71	42.52	243.05	207.80
0.5	50.08	257.71	216.19	51.91	257.77	214.72
0.6	21.26	237.99	220.36	24.69	238.04	217.57
0.7	46.67	267.63	228.94	48.77	267.69	227.25
0.8	58.02	282.84	234.74	59.67	282.90	233.43
0.9	29.83	264.81	240.07	33.18	264.86	237.35
**Avg**	**50.88**	**254.96**	**212.77**	**53.01**	**255.02**	**211.08**

Furthermore, Δ*H*^≠^ and Δ*G*^≠^ of the activated complex increased with increasing *α* and temperature during thermal conversion. The variation in Δ*H*^≠^ could represent the energetic variance between the dehydration and dehydroxylation steps of kaolin.^37^ Besides, the positive values of Δ*H*^≠^ match the presence of endothermic peaks in the DTA curves. Additionally, the temperature dependence of Δ*G*^≠^ is revealing the stability of the activated complex during the dehydroxylation process. The positive values of Δ*G*^≠^ underline that the dehydroxylation of the –OH groups may not spontaneously happen at low temperatures.^83^ It is also visible that the second decomposition step looks more difficult compared to the first step. The decrease of Δ*H*^≠^ and ΔG^≠^ for conversion rates *α* > 0.8 might be explained by the achievement of a maximum dehydroxylation rate, resulting in the formation of bonds during the change of the coordination of Si and Al atoms from the transformation of the octahedral layers.^84^ Furthermore, the resulting metakaolin has already reached its maximum delamination and the formed phase is a more reactive amorphous structure.^19^

**Fig. 9:**
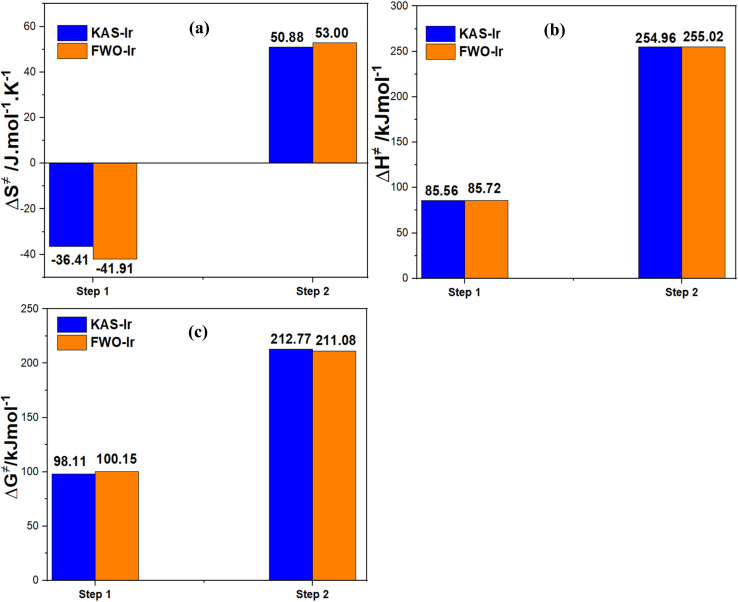
Bar charts of the average thermodynamic parameters Δ*S*^≠^(a), Δ*H*^≠^(b) and Δ*G*^≠^(c) of the conversion of cameroonian kaolin to metakaolin derived using *E*_a_ derived from either KAS-Ir or FWO-Ir methods.

For an easy overview, the average values of Δ*S*^≠^, Δ*H*^≠^ and Δ*G*^≠^ for the dehydration and dehydroxylation of kaolin are displayed as bar charts in Fig. 9 (left, middle and right). The chart presents close values of Δ*S*^≠^, Δ*H*^≠^ and Δ*G*^≠^ for the KAS-Ir and the FWO-Ir model for both reaction steps with a deviation between the models of less than 5%, confirming the accuracy and tenability of the obtained values of *E*_a_ using the iterative procedure and the most probable mechanism functions. Similar results were reported by Ptáček *et al.*, 2014.^35^

## 5 Conclusions

The thermal conversion of cameroonian kaolin to metakaolin was performed at *β =* 5, 20 and 40 K min^−1^. It was observed that the thermal conversion of kaolin to metakaolin occurred in two steps: a pre-dehydroxylation step (dehydration) at lower temperatures and a dehydroxylation step at higher temperatures. During the heat treatment high heating rates hindered the full conversion of kaolin and the metakaolin obtained (samples MK-(20) and MK-(40)) was found to be less amorphous.

The combination of the MHR method with the Coats-Redfern model allowed to elucidate the most probable mechanism function *g*(*α*) of the kaolin conversion. Our thermokinetic analysis showed that kaolin dehydroxylation was controlled by random nucleation and subsequent growth mechanisms (G_4_) and a chemical reaction of second order (F_2_). The iterative procedure with KAS-Ir and FWO-Ir approved to be well suited and attractive to examine the thermokinetic parameters *E*_a_ and *A* and thermodynamic variables Δ*S*^≠^, Δ*H*^≠^ and Δ*G*^≠^ of the kaolin conversion process. The differential methods of Kissinger and Ozawa were found to be inadequate.

Δ*H*^≠^ and Δ*G*^≠^ of the activated complex increased with increasing *α* and temperature during thermal conversion. The variation in Δ*H*^≠^ could represent the energetic variance between the dehydration and dehydroxylation steps of kaolin. The quality of the product, energy consumption, processing time, and consequently, industrial profitability are all directly affected by adjusting the heating rate during the thermal conversion of kaolin. The obtained metakaolin could be suitable for the synthesis of zeolite.

## Author contributions

Cyrille Ghislain Fotsop: writing – original draft, visualization, validation, resources, methodology, investigation, formal analysis, data curation, conceptualization. Alexandra Lieb: validation, supervision, investigation, formal analysis, conceptualization, data curation, writing – review & editing. Franziska Scheffler: validation, supervision, resources, funding acquisition, project administration, conceptualization.

## Conflicts of interest

The authors declare no conflict of interest.

## Supplementary Material

RA-015-D5RA05149E-s001

## Data Availability

Data will be made available on request. Supplementary information is available. See DOI: https://doi.org/10.1039/d5ra05149e.
