# Supplementary material for: Elucidation of the thermo-kinetics of the thermal decomposition of cameroonian kaolin: mechanism, thermodynamic study and identification of its by-products
Source: RSC Adv. 2025 Sep 8;15(39):32172–87. doi: 10.1039/d5ra05149e (PMC12415547; doi:10.1039/d5ra05149e)
Supplement: RA-015-D5RA05149E-s001 [file RA-015-D5RA05149E-s001.pdf]

Supplementary Information (SI) for RSC Advances.
This journal is © The Royal Society of Chemistry 2025

Electronic Supporting Information

Elucidation of the thermo-kinetics of the thermal decomposition of cameroonian kaolin: Mechanism, thermodynamic study and identification of its by-products

Cyrille Ghislain Fotsop[a], Alexandra Lieb[a], Franziska Scheffler[a]

[a] Otto-von-Guericke-University Magdeburg, Chemical Institute Industrial Chemistry, Universitätsplatz 2, 39106, Magdeburg, Germany

Equations

Degree of dehydroxylation (D_d) [1]

$$D_d = 1 - \left(\frac{M_m - M_T}{M_m}\right) \quad \text{(Eq. S1)}$$

Where M_T and M_m represent the mass loss and the maximum loss in weight% at a certain temperature, respectively.

Tables

Tab. S1: Algebraic expressions of functions $g(\alpha)$, $f(\alpha)$ and its corresponding mechanism [15,16]

No	Symbol	$g(\alpha)$	$f(\alpha)$	Rate-determining mechanism
**1. Chemical process or mechanism - non-invocative equations**				
1	$F_{1/3}$	$1-(1-\alpha)^{2/3}$	$3/2\,(1-\alpha)^{1/3}$	Chemical reaction
2	$F_{3/4}$	$1-(1-\alpha)^{1/4}$	$4\,(1-\alpha)^{3/4}$	Chemical reaction
3	$F_{3/2}$	$(1-\alpha)^{-1/2}-1$	$2\,(1-\alpha)^{3/2}$	Chemical reaction
4	F_2	$(1-\alpha)^{-1}-1$	$(1-\alpha)^2$	Chemical reaction
5	F_3	$(1-\alpha)^{-2}-1$	$1/2\,(1-\alpha)^3$	Chemical reaction
6	F_4	$(1-\alpha)^{-3}-1$	$1/3\,(1-\alpha)^4$	Chemical reaction
7	G_1	$1-(1-\alpha)^2$	$1/2\,(1-\alpha)$	Chemical reaction
8	G_2	$1-(1-\alpha)^3$	$1/3\,(1-\alpha)^2$	Chemical reaction
9	G_3	$1-(1-\alpha)^4$	$1/4\,(1-\alpha)^3$	Chemical reaction
**2. Acceleration rate equations**				
10	$P_{3/2}$	$\alpha^{3/2}$	$(2/3)\,\alpha^{-1/2}$	Nucleation
11	$P_{1/2}$	$\alpha^{1/2}$	$2\,\alpha^{1/2}$	Nucleation
12	$P_{1/3}$	$\alpha^{1/3}$	$3\,\alpha^{2/3}$	Nucleation
13	$P_{1/4}$	$\alpha^{1/4}$	$4\,\alpha^{3/4}$	Nucleation
14	E_1	α	$\ln\alpha$	Nucleation
**3. Sigmoidal rate equations or random nucleation and subsequent growth**				
15	A_1, F_1	$-\ln(1-\alpha)$	$1-\alpha$	Assumed random nucleation and its subsequent growth
16	$A_{3/2}$	$[-\ln(1-\alpha)]^{2/3}$	$3/2\,(1-\alpha)\,[-\ln(1-\alpha)]^{1/3}$	Assumed random nucleation and its subsequent growth
17	A_2	$[-\ln(1-\alpha)]^{1/2}$	$2\,(1-\alpha)\,[-\ln(1-\alpha)]^{1/2}$	Assumed random nucleation and its subsequent growth
18	A_3	$[-\ln(1-\alpha)]^{1/3}$	$3\,(1-\alpha)\,[-\ln(1-\alpha)]^{2/3}$	Assumed random nucleation and its subsequent growth
19	A_4	$[-\ln(1-\alpha)]^{1/4}$	$4\,(1-\alpha)\,[-\ln(1-\alpha)]^{3/4}$	Assumed random nucleation and its subsequent growth
20	G_4	$[-\ln(1-\alpha)]^2$	$1/2\,(1-\alpha)\,[-\ln(1-\alpha)]^{-1}$	Assumed random nucleation and its subsequent growth
21	G_5	$[-\ln(1-\alpha)]^3$	$1/3\,(1-\alpha)\,[-\ln(1-\alpha)]^{-2}$	Assumed random nucleation and its subsequent growth
22	G_6	$[-\ln(1-\alpha)]^4$	$1/4\,(1-\alpha)\,[-\ln(1-\alpha)]^{-4}$	Assumed random nucleation and its subsequent growth
23	Au	$\ln\alpha/(1-\alpha)$	$\alpha/(1-\alpha)$	Branching nuclei
**4. Deceleration rate equations**				
**4.1 Phase boundary reaction**				
24	R_1, F_0, P_1	α	$(1-\alpha)^0$	Contracting disk
25	R_2, $F_{1/2}$	$1-(1-\alpha)^{1/2}$	$2\,(1-\alpha)^{1/2}$	Contraction cylinder (cylindrical symmetry)
26	R_3, $F_{2/3}$	$1-(1-\alpha)^{1/3}$	$3\,(1-\alpha)^{2/3}$	Contraction cylinder (spherical symmetry)
**4.2 Based on the diffusion mechanism**				
27	D_1	α^2	$1/(2\alpha)$	One-dimensional diffusion
28	G_7	$[1-(1-\alpha)^{1/2}]^{1/2}$	$4\{(1-\alpha)\,[1-(1-\alpha)^{1/2}]\}^{1/2}$	Two-dimensional diffusion

29	D_2	$\alpha + (1-\alpha)\ln(1-\alpha)$	$[-\ln(1-\alpha)]^{-1}$	Two-dimensional diffusion
30	D_3	$[1-(1-\alpha)^{1/3}]^2$	$3/2(1-\alpha)^{2/3}[1-(1-\alpha)^{1/3}]^{-1}$	Three-dimensional diffusion, spherical symmetry
31	D_4	$1-2/3\alpha-(1-\alpha)^{2/3}$	$3/2[(1-\alpha)^{-1/3}-1]^{-1}$	Three-dimensional diffusion, spherical symmetry
32	D_5	$[(1-\alpha)^{-1/3}-1]^2$	$3/2(1-\alpha)^{4/3}[(1-\alpha)^{-1/3}-1]^{-1}$	Three-dimensional diffusion
33	D_6	$[(1+\alpha)^{-1/3}-1]^2$	$3/2(1+\alpha)^{4/3}[(1+\alpha)^{-1/3}-1]^{-1}$	Three-dimensional diffusion
34	D_7	$1 + 2/3\alpha - (1+\alpha)^{+2/3}$	$3/2[(1+\alpha)^{-1/3}-1]^{-1}$	Three-dimensional diffusion
35	D_8	$[(1+\alpha)^{-1/3}-1]^2$	$3/2(1+\alpha)^{4/3}[(1+\alpha)^{-1/3}-1]^{-1}$	Three-dimensional diffusion
36	G_8	$[1-(1-\alpha)^{-1/3}]^{1/2}$	$6(1-\alpha)^{2/3}[1-(1-\alpha)^{1/3}]^{1/2}$	Three-dimensional diffusion

Tab. S2: Activation energy E_a, R^2 and pre-exponential factor A calculated using Kissinger and Ozawa equations and T_m based on DTG peaks

Equation	Step 1			Step 2		
	E_a/kJmol^{-1}	A/min^{-1}	R^2	E_a/kJmol^{-1}	A/min^{-1}	R^2
**Kissinger**	73.08	$5.39\cdot10^{12}$	0.92502	254.51	$4.94\cdot10^{16}$	0.99808
**Ozawa**	93.61	$4.06\cdot10^{14}$	0.95517	261.83	$6.77\cdot10^{16}$	0.99838

Tab. S3: Activation energy E_a and R^2 calculated for step 1 and 2 using KAS and FWO methods without the iterative procedure

		Step 1			Step 2		
	α	E_a/kJmol^{-1}	R^2	A/min^{-1}	E_a /kJmol^{-1}	R^2	A/min^{-1}
KAS	0.1	55.86	0.99996	-	257.20	0.99996	-
	0.2	68.04	0.99988	-	231.26	0.99988	-
	0.3	73.46	0.99760	-	245.40	0.99760	-
	0.4	74.46	0.98585	-	240.38	0.98585	-
	0.5	75.41	0.99979	-	254.94	0.99979	-
	0.6	86.84	0.99979	-	235.04	0.99979	-
	0.7	89.98	0.99625	-	264.47	0.99625	-
	0.8	90.66	0.98092	-	279.50	0.98092	-
	0.9	80.65	0.99357	-	261.11	0.99357	-
**Average**		**77.26**			**252.15**		
FWO	0.1	94.36	0.99254	-	262.64	0.99996	-
	0.2	95.72	0.99881	-	239.09	0.99992	-
	0.3	94.97	0.99802	-	252.21	0.99797	-
	0.4	93.49	0.99999	-	247.78	0.98799	-
	0.5	93.06	0.99273	-	261.94	0.98799	-
	0.6	102.76	0.99105	-	243.27	0.99980	-
	0.7	104.63	0.99907	-	271.73	0.99682	-
	0.8	104.74	0.99976	-	286.39	0.98360	-
	0.9	94.75	0.99979	-	269.47	0.99459	-
**Average**		**97.61**			**259.39**		

Tab. S4: The α and β values for the plots of ln$g(\alpha)$ versus lnβ of the conversion of cameroonian kaolin

	Step 1	Step 2
**β/Kmin^{-1}**	**α at T_1=72.908 °C**	**α at (T_2=555.884 °C)**
**5**	0.622	0.617
**20**	0.403	0.341
**40**	0.285	0.166

Tab. S5: Symbols of the algebraic expressions (detailed expressions in Tab. S1) of the function $g(\alpha)$, R^2 and slopes obtained using the Coats Redfern model

Function	β(Kmin^{-1})	$g(\alpha)$	R^2	Slope
**F2**	5		0.96688	-7.60852
	20	$(1-\alpha)^{-1}-1$	0.96961	-7.82648
	40		0.96911	-7.73141
**F3**	5		0.92880	-11.13041
	20	$(1-\alpha)^{-2}-1$	0.92925	-11.42803
	40		0.92808	-11.30480
**A1, F1**	5		0.97571	-4.77097
	20	$-\ln(1-\alpha)$	0.98340	-4.92411
	40		0.98406	-4.85174
**D1**	5		0.92778	-7.90818
	20	α^2	0.93711	-8.15711
	40		0.93845	-8.06816
**D2**	5		0.95357	-9.01555
	20	$\alpha+(1-\alpha)\ln(1-\alpha)$	0.96139	-9.29055
	40		0.96252	-9.19265
**D6**	5		0.93680	-8.43176
	20	$[(1+\alpha)^{-1/3}-1]^2$	0.94328	-8.66963
	40		0.94513	-8.61855
**D8**	5		0.87874	-6.01877
	20	$[(1+\alpha)^{-1/3}-1]^2$	0.89079	-6.21999
	40		0.89251	-6.14570

Scheme

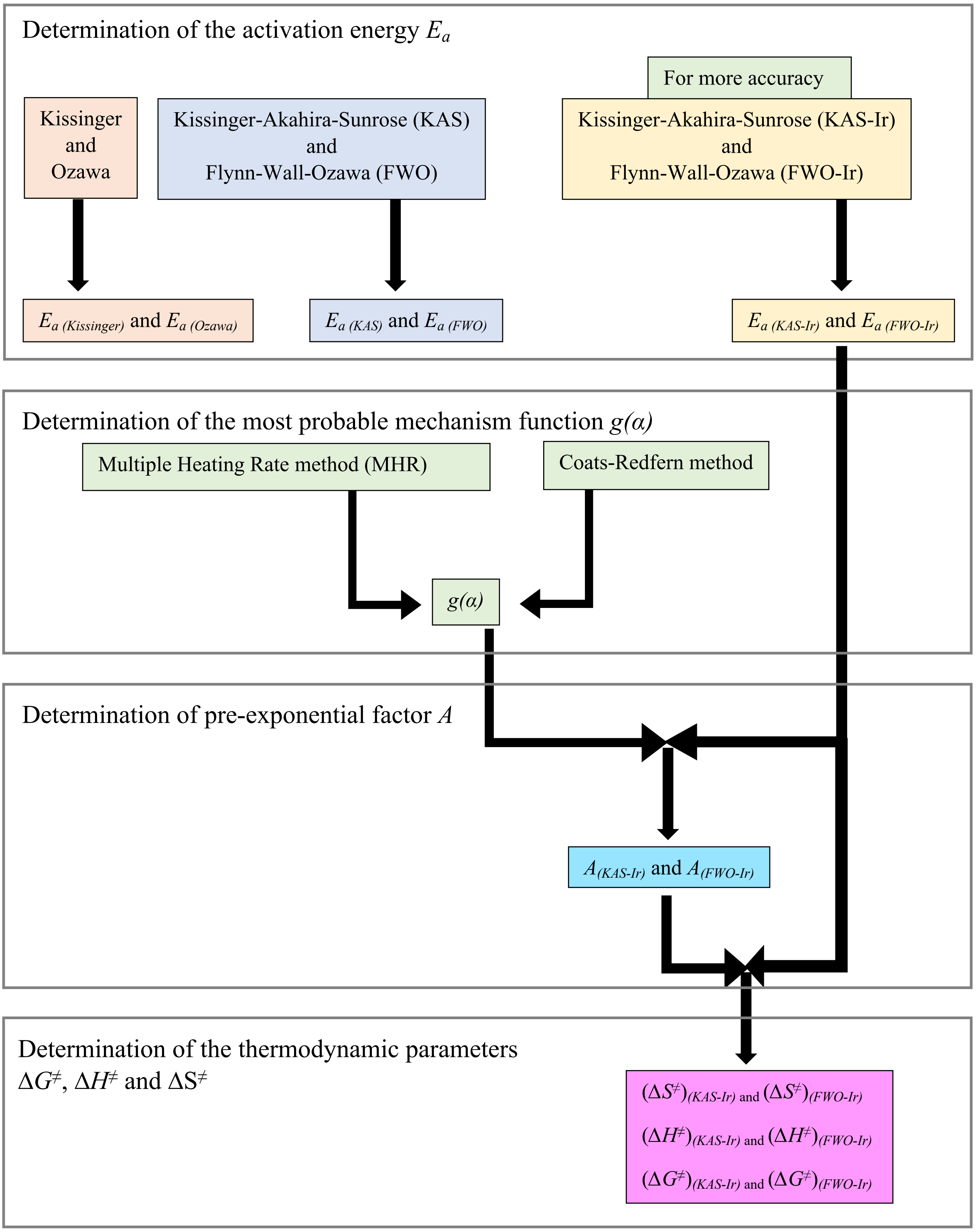

Scheme S1: Overview of the used methods and models and the calculation pathways to determine accurate values for E_a, A, $g(\alpha)$ and the thermodynamic parameters $\Delta G^{\neq}$, $\Delta H^{\neq}$ and $\Delta S^{\neq}$.

Figures

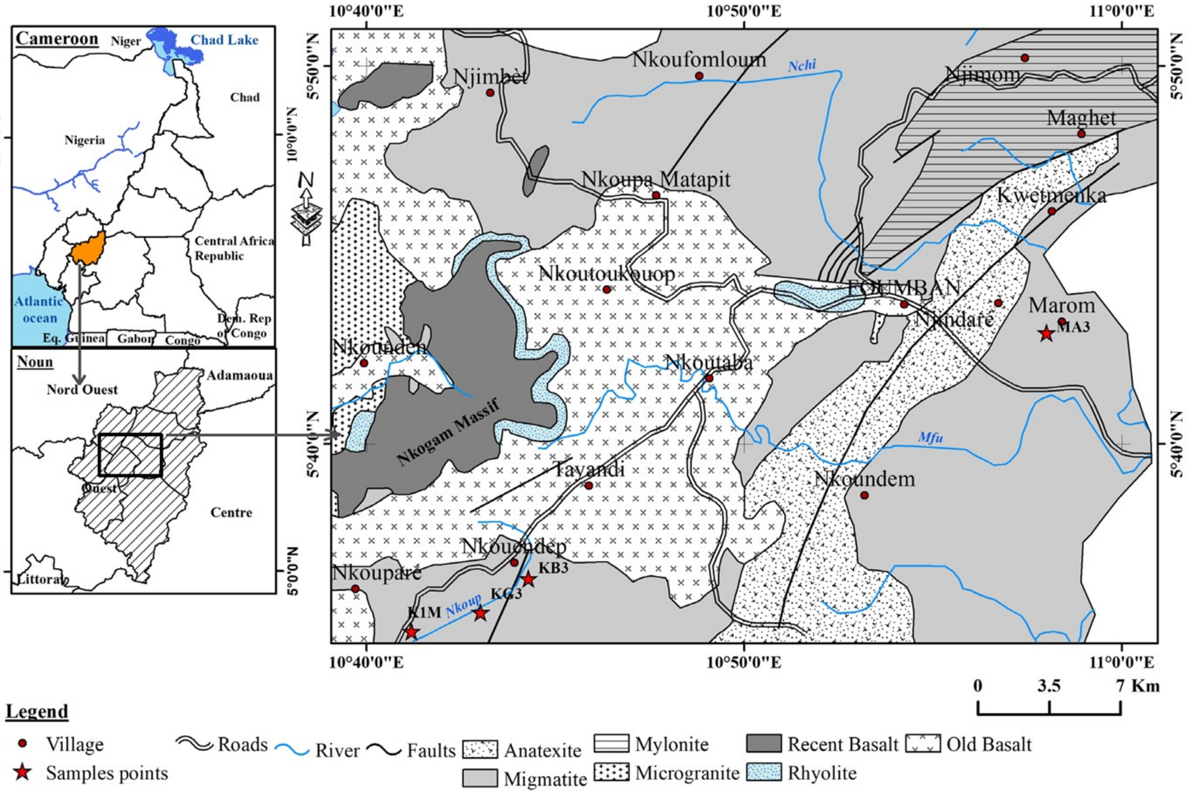

Fig. S1: Location of the collected samples on the geological map of West Region of Cameroon (Foumban) [4].

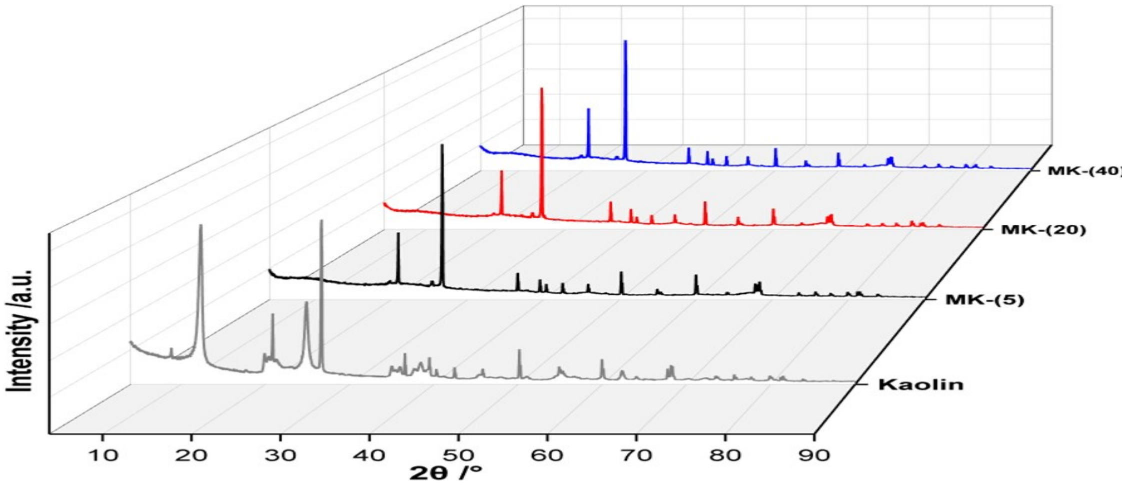

Fig. S2: Cascade plot of the diffraction patterns of the cameroonian kaolin and the derived metakaolin samples MK-(5), MK-(20) and MK-(40), which have been recorded using a PANalytical Empyrean diffractometer with Cu $K_{\alpha1+\alpha2}$ radiation. The plots clearly show the amorphous background representing the metakaolin.

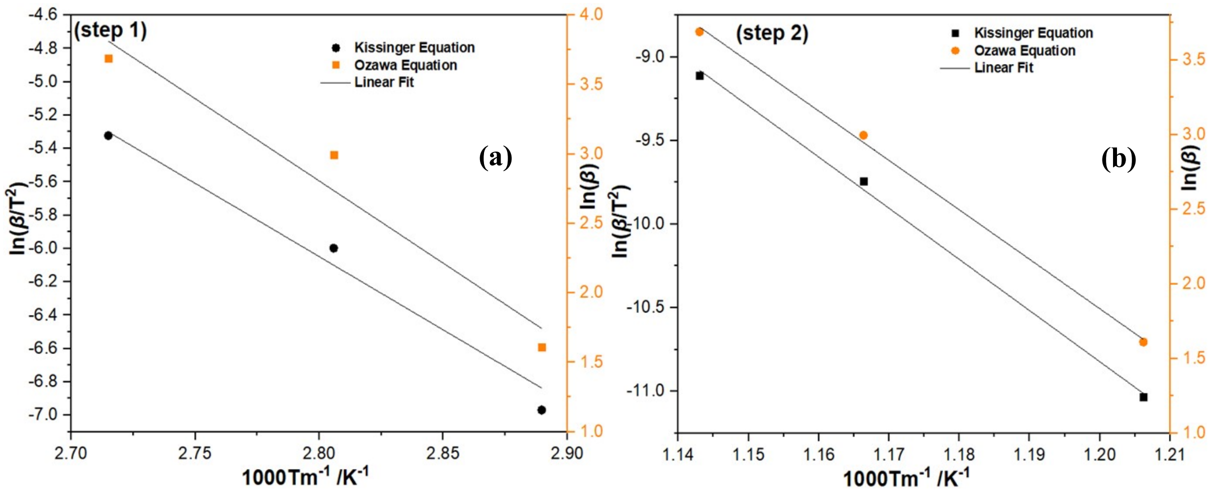

Fig. S3: Thermokinetics of kaolin for step 1 (a) and step 2 (b) using Kissinger and Ozawa differential methods based on DTG data. The E_a for the respective steps was determined from the slope of the linear fits.

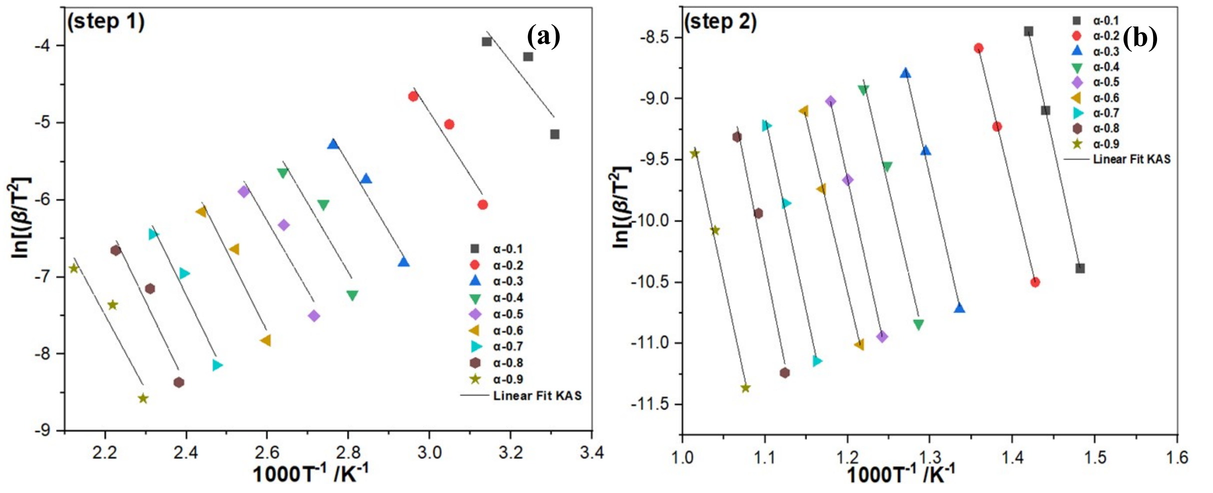

Fig. S4: Thermokinetics of kaolin for step 1 (a) and step 2 (b) using KAS methods based on DTG data without the iterative procedure.

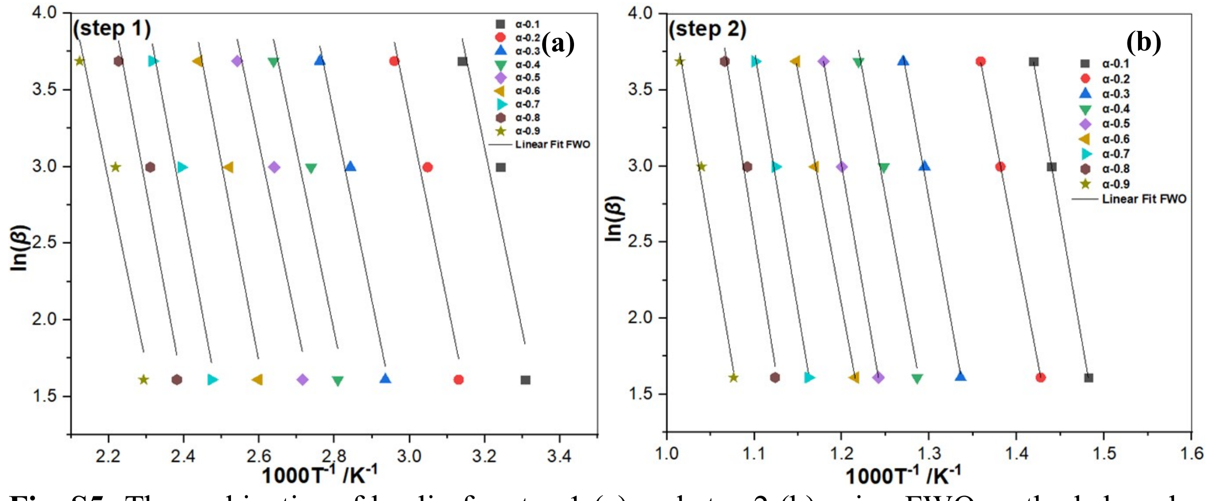

Fig. S5: Thermokinetics of kaolin for step 1 (a) and step 2 (b) using FWO methods based on DTG data without the iterative procedure.

References

[1] M. Irfan Khan *et al.*, « The pyrolysis kinetics of the conversion of Malaysian kaolin to metakaolin », *Appl. Clay Sci.*, vol. 146, p. 152-161, sept. 2017, doi: 10.1016/j.clay.2017.05.017.

[2] H. Jiang, J. Wang, S. Wu, B. Wang, et Z. Wang, « Pyrolysis kinetics of phenol – formaldehyde resin by non-isothermal thermogravimetry », *Carbon*, vol. 48, n° 2, p. 352-358, 2009, doi: 10.1016/j.carbon.2009.09.036.

[3] C. Sronsri, P. Noisong, et C. Danvirutai, « Isoconversional kinetic, mechanism and thermodynamic studies of the thermal decomposition of NH4Co0.8Zn0.1Mn0.1PO4·H2O », *J. Therm. Anal. Calorim.*, vol. 120, n° 3, p. 1689-1701, juin 2015, doi: 10.1007/s10973-015-4471-x.

[4] A. M. Nkalih, P. Pilate, R. F. Yongue, A. Njoya, et N. Fagel, « Suitability of Foumban Clays (West Cameroon) for Production of Bricks and Tiles », *J. Miner. Mater. Charact. Eng.*, vol. 06, n° 02, p. 244-256, 2018, doi: 10.4236/jmmce.2018.62018.
